# Cisplatin and renal injury; current concepts

**DOI:** 10.12861/jrip.2013.28

**Published:** 2013-09-01

**Authors:** Hamid Nasri

**Affiliations:** ^1^Department of Nephrology, Division of Nephropathology, Isfahan University of Medical Sciences, Isfahan, Iran

**Keywords:** Cisplatin, Renal failure, Nephrotoxicity

**Implication for health policy/practice/research/medical education:**

Cisplatin gives side-effects such as kidney injury which is dose-dependent, limited its use.In recent years much attention has been directed toward the gender difference in cisplatin kidney damage. Indeed, there are sharp sex-dependent differences in reaction rates and the probability of side effects in individuals treated with chemotherapy. Howeverinvestigations regarding sex difference in cisplatin kidney damage is scares and more needs needs to find the clinical significance of gender on renal failure induced by cisplatin.

Cisplatin [cis-diammine dichloride platinum (II) (CDDP)] a platinum-based chemotherapy agent used to treat some types of cancers in pediatric and adults (1). However, due to its accumulation in kidney, nephrotoxicity is the most common side-effect of cisplatin treatment (1,2).Treatment with cisplatin induces the inflammatory mechanisms, which leads to a reduction in the antioxidant levels, leading to a failure of the antioxidant protection against free-radical damage generated by antitumor drugs (3). Inflammation, endoplasmic reticulum (ER) stress and oxidative stress, contribute to cisplatin-induced kidney toxicity. In turn, cisplatin disturbs the oxidant/antioxidant balance and its nephropathy is closely associated with an increase in lipid peroxidation (2-4). In was found that, oxidative stress has been incriminated to play a role in the development of kidney disease indirectly by promoting hypertension and atherosclerosis or directly by inducing glomerular injury and kidney ischemia (3-5). It consequences from a decrease of natural cell antioxidant capacity or an increased quantity of reactive oxygen species in kidney (2-4). The oxidative stress, brought by cisplatin in the kidney was partially inhibited by antioxidant therapy using such as vitamin C or E, flavonoids, superoxide dismutase, glutathione and selenium (2-4). Hence various substances, have been examined to find their efficacy to reduce cisplatin-induced kidney injury (3-5). The kidney injury effects of cisplatin are demonstrated by a decrease in creatinine clearance and electrolyte disturbances, mainly hypomagnesemia, largely due to the acute cytotoxic properties of cisplatin on distal and proximal tubules (1-5). In recent years, there has been, there was a trend to identify urinary biomarkers of kidney toxicity as noninvasive measurements with larger sensitivity and specificity than traditional biomarkers, such as serum creatinine and blood urea nitrogen (2-5). In fact a variety of novel urinary biomarkers have been known and partially authorized for use as the markers for renal injury in cisplatin kidney toxicity (2-5). These novel biomarkers are urinary albumin, kidney injury molecule-1 and plasma cystatin C, alongside the traditional biomarkers of plasma creatinine, urea and urinary total protein, glucose and n-acetyl-beta-d-glucosaminidase (3-6). It is possible that in the near future, some of these urinary biomarkers may be suggested to more precisely show the cisplatin renal damage and accepted as the biomarkers of cisplatin kidney damage (3-6). Another aspect of cisplatin kidney damage is gender difference of cisplatin nephrotoxicity (7). It is documented that chronic kidney diseases are gender related. Indeed, there are sharp gender-dependent differences in reaction rates and the probability of side effects in individuals treated with chemotherapy (6-8). Various chemotherapy protocols containing cisplatin lead to a better response rate in women without increasing toxicity, while others drugs like 5-fluorouracil only increases toxicity, but do not improve response rates in female gender (6-8). To find the cisplatin kidney damage, enhanced urinary sodium excretion in male but not in female rats was found during cisplatin therapy (7,8). While recent studied explained a cisplatin gender related of toxicity due to an unknown mechanism. However, it should be pointed out that, studies of gender difference in cisplatin nephropathy is scares and more investigations needs to find the clinical significance of sex on renal damage induced by cisplatin, also, more study on the biomarkers of acute kindly injury during cisplatin toxicity suggests to better find the acute kidney injury of cisplatin and effectively abolishes damages to the kidneys and decrease morbidity of these patients (7,8).

## Author’s contribution

HN is the single author of the manuscript.

## Conflict of interests

The author declared no competing interests.

## Ethical considerations

Ethical issues (including plagiarism, data fabrication, double publication) have been completely observed by the author.

## Funding/Support

None.
